# Defect-Induced Efficient Heteroepitaxial Growth of Single-Wall Carbon Nanotubes @ Hexagonal Boron Nitride Films

**DOI:** 10.3390/ma16051864

**Published:** 2023-02-24

**Authors:** Changping Yu, Lili Zhang, Gang Zhou, Feng Zhang, Zichu Zhang, Anping Wu, Pengxiang Hou, Huiming Cheng, Chang Liu

**Affiliations:** 1 Shenyang National Laboratory for Materials Science, Institute of Metal Research, Chinese Academy of Sciences, 72 Wenhua Road, Shenyang 110016, China; 2 School of Materials Science and Engineering, University of Science and Technology of China, Hefei 230026, China; 3 Shi-changxu Innovation Center for Advanced Materials, Institute of Metal Research, Chinese Academy of Sciences, Shenyang 110016, China; 4 Shenzhen Institute of Advanced Technology, Chinese Academy of Sciences, Shenzhen 518055, China

**Keywords:** carbon nanotubes, hexagonal boron nitride, defect, derivatives

## Abstract

Carbon nanotube-based derivatives have attracted considerable research interest due to their unique structure and fascinating physicochemical properties. However, the controlled growth mechanism of these derivatives remains unclear, and the synthesis efficiency is low. Herein, we proposed a defect-induced strategy for the efficient heteroepitaxial growth of single-wall carbon nanotubes (SWCNTs)@hexagonal boron nitride (h-BN) films. Air plasma treatment was first performed to generate defects on the wall of SWCNTs. Then, atmospheric pressure chemical vapor deposition was conducted to grow h-BN on the surface of SWCNTs. Controlled experiments combined with first-principles calculations revealed that the induced defects on the wall of SWCNTs function as nucleation sites for the efficient heteroepitaxial growth of h-BN.

## 1. Introduction

Carbon nanotubes (CNTs) with excellent physicochemical properties are widely used in nanoelectronics, catalysis, energy storage, sensing [1,2,3], etc. In addition, single-wall carbon nanotubes (SWCNTs) have been considered a promising template for the growth of CNT-based derivatives [4,5,6]. Owing to the similar sp^2^ hybridization of CNTs and hexagonal boron nitride (h-BN), CNT@h-BN derivatives and their macrostructures with novel synergistic physiochemical properties have been prepared and intensively studied [7,8,9,10].

Compared with bare CNTs, CNT@h-BN exhibits superior thermal and chemical stability [11,12,13], higher thermal conductivity [14,15,16], and stronger mechanical robustness [17,18]. Chemical vapor deposition (CVD) has been used to prepare CNT@h-BN with controllable layer number, crystallinity, and coaxiality [19,20,21]. Perfect CNT-based 1D van der Waals heterostructures [22,23,24,25] were obtained by Xiang et al. using a low-pressure CVD method, and they found that isolated ultraclean SWCNTs were essential to achieve an open-end growth mode. However, the growth efficiency (the ratio of the SWCNT surfaces that is coated with h-BN) was low, possibly because the structural defects and carbonaceous impurities on the wall of SWCNTs may interrupt the continuous growth of the BN layer [26,27]. On the other hand, Zheng et al. reported that amorphous carbon on the wall of SWCNTs might serve as nucleation sites for the growth of h-BN [28]. Overall, the detailed growth mechanism of CNT@h-BN is still unclear, and its growth efficiency needs to be improved.

In this study, we developed a defect-induced heteroepitaxial method to efficiently synthesize SWCNT@h-BN films. Freestanding high-quality SWCNT films prepared by the floating catalysts CVD (FCCVD) method were used as a growth template. Air plasma treatment was performed to introduce defects on the tube walls that serve as nucleation sites in the following heteroepitaxial growth of h-BN by atmospheric pressure CVD (APCVD). Macroscopic SWCNT@h-BN films were obtained, and their growth mechanism was elucidated by combining experimental investigation and first-principles calculations. The microstructure of the SWCNT@h-BN films was tuned by adjusting the density of defects on the SWCNT template. Defects were controllably introduced by air plasma, and more sites were supplied for the nucleation of h-BN. Furthermore, the sp^2^ hybridization connected SWCNT and h-BN increased the interaction and triggered efficient epitaxial growth. Both controllability and efficiency were improved significantly. Our work provides an efficient approach to the preparation of SWCNT-based derivative macrostructures, which may facilitate their applications in a wide range of areas.

## 2. Materials and Methods

### 2.1. Experimental Setup and Conditions

SWCNT films were synthesized by an injection FCCVD method [29]. Freestanding SWCNT films were collected on a filter membrane and transferred to a molybdenum frame (1 mm in thickness) for the following growth of h-BN and subsequential characterizations.

Before the CVD growth of h-BN, air plasma treatment was performed to controllably introduce defects on the wall of SWCNTs [30]. Briefly, a plasma cleaner (Harrick PDC-002, Ithaca, State of New York, USA) with a gas flow mixer (Harrick PDC-FMG-2, Ithaca, NY, USA) was evacuated to less than 50 Pa, and the SWCNT film was exposed to air plasma for 0~150 s with a power supply of 7 W. APCVD was performed to synthesize SWCNT@h-BN using borane-tert-butylamine complex (C_4_H_14_BN, 97% Sigma-Aldrich, Burlington, MA, USA) as a precursor. A schematic of the APCVD setup is shown in Figure S1. Plasma-treated SWCNT film was placed at the center of a quartz tube reactor inserted into a horizontal tubular furnace (1 inch), 30 mg C_4_H_14_BN was loaded upstream and heated to 90 °C by a heating belt, and 300 sccm of Ar was introduced as a carrier gas. The reaction temperature was set to be 1000 °C, and the growth time was adjusted in the range of 5–180 min. After growth, the CVD furnace was cooled down to ambient temperature naturally under the protection of an Ar flow.

### 2.2. Samples Characterization

Transmission electron microscopy (TEM, FEI Tecnai G^2^ F20, Hillsboro, Oregon, USA, operated at 200 kV) was used to characterize the microstructure of the SWCNTs and SWCNT@h-BN films. High-angle annular dark-field scanning transmission electron microscopy (HAADF-STEM) image and electron energy loss spectroscopy (EELS) mapping of SWCNT@h-BN were acquired using a spherical aberration-corrected TEM (FEI Titan Cubed Themis G2300, Hillsboro, Oregon, USA, operated at 300 kV).

The Raman spectra of SWCNTs and SWCNT@h-BN films were collected by micro-Raman spectroscopy (Witec, ALPHA300R, Ulm, Baden-Württemberg Germany, equipped with 532 and 633 nm lasers). The ratios of I_G_/I_D_ ratio were randomly collected from five points for each sample and calculated by averaging these data. Fourier transform infrared (FT-IR) spectra of all samples were collected from spectrometer (Bruker Tensor 27, Karlsruhe, Baden-Württemberg, Germany) in the range of 400–4000 cm^−1^. X-ray photoelectron spectroscopy (XPS, ESCALABXi+, Waltham, MA, USA, operated at 15 kV and 150 W) was used to analyze the chemical composition of surface layers using a pass energy of 50 eV with an energy step size of 0.1 eV. The collected spectra were calibrated by the standard C 1s peak located at 284.6 eV.

### 2.3. First-Principles Calculations

First-principles calculations based on the density functional theory implemented in the Vienna ab initio simulation package (VASP) [31,32] with the projector augmented wave (PAW) [33,34] method and the Perdew–Becke–Erzenhof (PBE) [35] exchange-correlation functional was adopted to reveal the growth mechanism of h-BN on defect sites of SWCNT. The calculations were performed in the 270-atom supercells of 5 BN and L10 SWCNT formed by 260 carbon atoms, with a cut-off plane wave energy of 550 eV and a 1 × 1 × 1 k-point grid.

## 3. Results and Discussion

### 3.1. Growth and Characterization of SWCNT@h-BN Film

**Figure 1:**
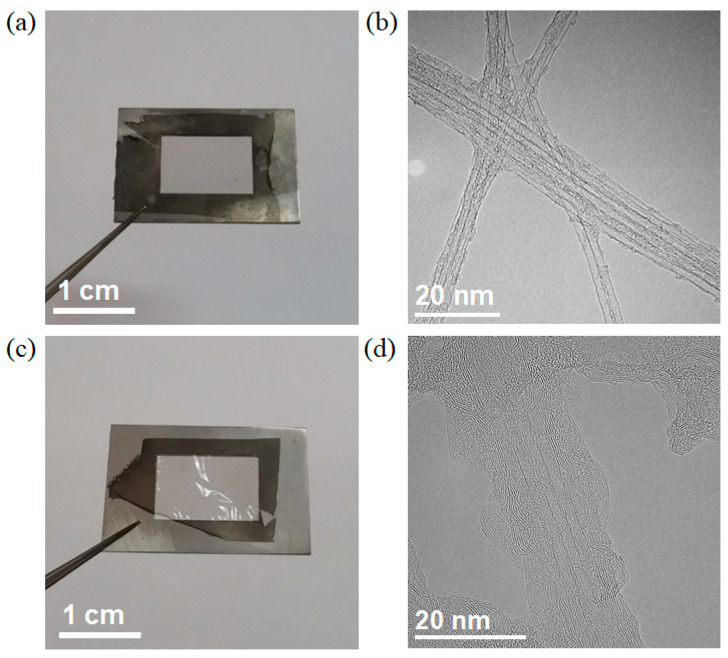
(**a**,**c**) Optical and (**b**,**d**) TEM images of a SWCNT film (**a**,**b**) before and (**c**,**d**) after CVD growth of h-BN.

Figure 1a shows a typical optical image of an SWCNT film prepared by injection FCCVD. The SWCNT film was transparent and flexible. The brightfield image obtained in TEM mode (Figure 1b) shows that the films were composed of randomly distributed SWCNT bundles. The diameters of these bundles were distributed in the range of 5–20 nm. The tube walls were straight and free of large-size impurities, indicating a high quality of the SWCNT template. After CVD growth of h-BN, the film continuously remains freestanding but became glossier (Figure 1c). As shown in Figure 1d, SWCNT bundles were fully wrapped by multi-layered substances. Though the coating layer was not totally coaxial with the tube wall, the thickness of the coating was basically uniform.

**Figure 2:**
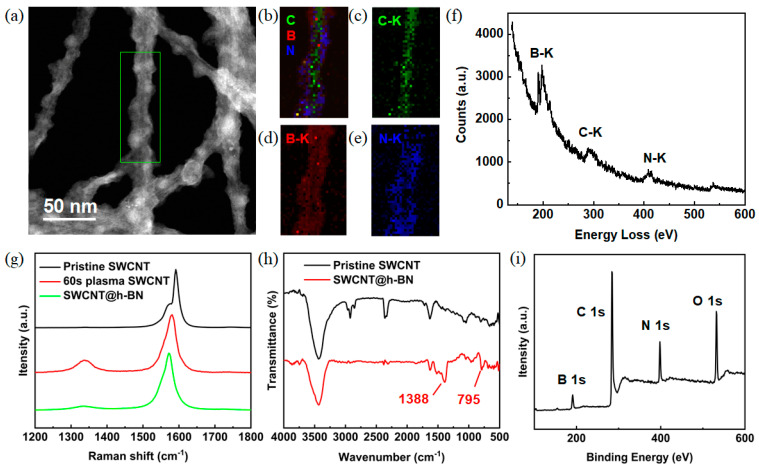
Structural characterization of SWCNT@h-BN. (**a**) ADF image, (**b**–**e**) EELS mapping, and (**f**) EELS spectrum of SWCNT@h-BN. (**g**) G and D bands Raman spectra of pristine SWCNTs, plasma-treated SWCNTs, and SWCNT@h-BN. (**h**) FT-IR spectra of original SWCNT and SWCNT@h-BN. (**i**) XPS spectrum of SWCNT@h-BN.

To characterize the chemical composition of the layer structure coated on the SWCNT surface, EELS was acquired in the STEM mode with an energy resolution of 2 eV. The low-magnification annular dark-field (ADF) image (Figure 2a) showed bead-like materials coated on the SWCNT bundle. EELS elemental mapping collected from the green rectangle of Figure 2a showed that B and N homogeneously distribute on the surface of SWCNTs (Figure 2b–e). The inner layer was composed of carbon while the outer layer was BN, indicating the formation of a core-shell-structured SWCNT@BN. In Figure 2f, the ionization edges around 198, 288, and 408 eV corresponded to the K-shell ionization edges of boron, carbon, and nitrogen, respectively. For further analysis, the characteristic peaks located at 190.5 eV and 197.8 eV were respectively attributed to the π* and σ* bonds from the B-K edge, confirming the sp^2^ bond of h-BN [36,37]. The sp^2^ hybridization of SWCNT and h-BN increased the interaction and triggered efficient epitaxial growth. Additionally, the formation of sp^2^ hybridization would improve the capacity of phonon transformation, realizing excellent thermal conductivity.

Raman spectroscopy and FT-IR spectroscopy were performed to characterize the phase of materials coated on the SWCNT surface. Radial breathing mode (RBM) Raman peaks collected from pristine SWCNTs, plasma-treated SWCNTs, and SWCNT@h-BN are shown in Figure S2a, demonstrating the existence of SWCNTs before and after the growth of h-BN. Typical Raman spectra of pristine SWCNTs, plasma-treated SWCNTs, and SWCNT@h-BN are shown in Figure 2g. The peak intensity of the G/D band (I_G_/I_D_) is widely used to evaluate the crystallinity of SWCNTs. The I_G_/I_D_ of the original SWCNT film was 133, indicating its high quality. After air plasma treatment, the I_G_/I_D_ value decreased to 4.6, demonstrating that abundant defects were introduced. Since the location of the D band (around 1350 cm^−1^) for sp^3^ carbon and E_2g_ (around 1366 cm^−1^) peak for h-BN is very close [19], to distinguish the contributions from SWCNT and h-BN, Gaussian fitting was utilized to deconvolve the peak around 1360 cm^−1^ (Figure S2b), and the fitted lines represent individual peaks of the D band of SWCNT and the E_2g_ mode of h-BN. The green peak at 1366 cm^−1^ was attributed to the E_2g_ of h-BN, indicating the existence of the h-BN phase, while the red peak around 1350 cm^−1^ represented the fitted D band of SWCNTs. The peak intensity of the G/fitted D band collected from SWCNT@h-BN was 12.2, much higher than that of the plasma-treated SWCNTs, suggesting that some defects were healed during the h-BN growth process. The standard deviation of pristine SWCNTs, 60 s plasma-treated SWCNTs, and SWCNT@h-BN was calculated to be 6.51, 0.79, and 1.19, respectively. FT-IR spectrum was also collected from SWCNT films before and after h-BN growth (Figure 2h). The peaks around 1388 cm^−1^ and 795 cm^−1^ represented the in-plane stretching mode of BN and the out-of-plane bending mode of B-N-B, respectively, which indicates the existence of the B-N bond [14,17].

To characterize the elemental stoichiometry and bond characteristics of the SWCNT@h-BN, XPS measurement was also performed. As shown in Figure 2i, the peaks located at 284.6 eV, 190.8 eV, and 398.4 eV were assigned to the binding energy of C 1s, B 1s, and N 1s, respectively. Specifically, a peak splitting of C 1s (Figure S3a) with four peaks centered at 283.9 eV, 284.7 eV, 286 eV, and 288.5 eV were attributed to C-B, C-C, C-N, and C-O bonding, respectively, which indicates the formation of covalent bond between h-BN and SWCNT. Similar peak splitting was also observed for the peaks of B 1s and N 1s (Figure S3b,c).

### 3.2. Growth Mechanism of SWCNT@h-BN

**Figure 3:**
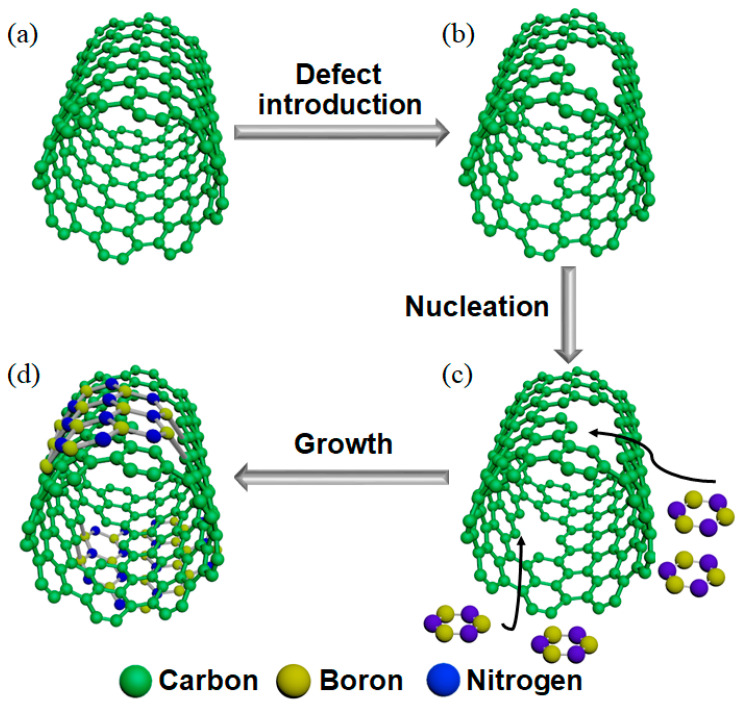
Schematic illustration of the defect-induced heteroepitaxial growth mechanism of SWCNT@h-BN.(**a**) Perfect SWCNT. (**b**) SWCNT with defects. (**c**) Atomic B and N adsorbed on the defects of SWCNT. (**d**) SWCNT coated by h-BN.

We proposed a defect-induced heteroepitaxial growth mechanism of SWCNT@h-BN, as shown in Figure 3. Ionized gas with high chemical reactivity was introduced to break the C-C covalent bonds, and vacancies and defects were generated on the wall of SWCNTs during the air plasma treatment (Figure 3a,b). The plasma-treated tube walls turned rough, and the distinct boundaries between the walls became blurred (Figure S4a). Then, atomic B and N decomposed from C_4_H_14_BN tended to adsorb on the defect sites and to form covalent bonds with C (Figure 3b,c). As the B and N atoms had a similar radius as that of C, BN would nucleate and start the heteroepitaxial growth process. Subsequently, multi-layered h-BN was formed and grown to form a tubular structure (Figure S4b) by using the wall of SWCNT as a “template” (Figure 3c,d).

To investigate the influence of growth parameters on the morphology of SWCNT@h-BN, we performed APCVD under different conditions. APCVD was first performed at 900 °C, 1000 °C, and 1100 °C for the growth of h-BN. As shown in Figure S5a–c, the morphologies of SWCNT@h-BN grown at different temperatures were similar, indicating the growth temperature in the range of 900–1100 °C had no apparent influence on the structure of SWCNT@h-BN. 

To verify the proposed defect-induced growth mechanism, we performed a control experiment by using SWCNTs without plasma treatment as a template. It was found that h-BN mostly nucleated at the junction of SWCNTs (Figure S6a) rather than from the middle of the tubes. This was because the junction of SWCNTs had a larger contact area with the BN precursor than the middle of the tube. After nucleation, h-BN began to grow and wrap the SWCNTs. However, amorphous carbon and other impurities would block the epitaxial growth of BN, leading to the formation of BN particles instead of a uniform crystalline h-BN layer (Figure S6b). Within the same growth time of 30 min, over 90% of surfaces of the plasma-treated SWCNTs were coated by h-BN through a defect-induced heteroepitaxial growth mode (Figure 1d), while the SWCNTs without defect introduction were only partially coated (~30%) (Figure S6b), indicating the significant efficiency achieved by defect-induced heteroepitaxial method.

Additionally, we investigated the effect of growth time on the growth of SWCNT@h-BN. Figure S7 shows TEM images of the SWCNT@h-BN obtained at different growth times of 5 min, 15 min, 30 min, and 120 min. We can observe that thin BN layers were homogeneously coated on the surface of SWCNT within a short growth time (Figure S7a,b). By prolonging the growth time, the thickness of the h-BN layer became thicker and thicker (Figure S7c,d).

**Figure 4:**
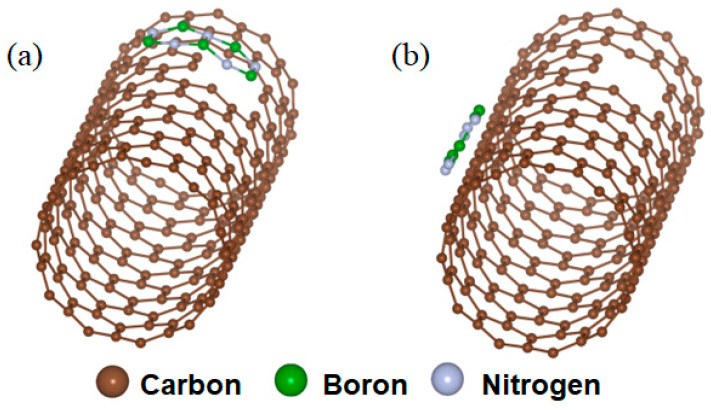
First-principles calculations of BN adsorption on the wall of SWCNTs (**a**) with defects and (**b**) without defects.

To elucidate the defect-induced heteroepitaxial growth mechanism, we performed first-principles calculations to further investigate the role of defects in the nucleation of h-BN. In order to simulate the SWCNT surface, 27 × 27 × 25.37 Å^3^ supercells were employed, and BN molecules were subsequently placed on the tube wall with defects, and with a perfect surface (Figure 4). We used one-dimensional periodic boundary conditions along the tube axis to simulate an infinitely long nanotube, and the length was 25.37 Å. Along the other two non-periodic directions, a vacuum region of ∼18 Å was taken into consideration. We defined the adsorption difference as ΔE = E_defect_ − E_perfect_, where E_defect_ is the energy of the model of BN molecules on the defect (Figure 4a), and E_perfect_ is the energy of the model of BN molecules on the perfect surface (Figure 4b). As calculated, the value of ΔE was −17.58 eV, on average, the energy difference contributed by each BN molecule was ~3.52 eV. Thus, the BN preferred to adsorb on the defect to achieve efficient heteroepitaxial nucleation.

**Figure 5:**
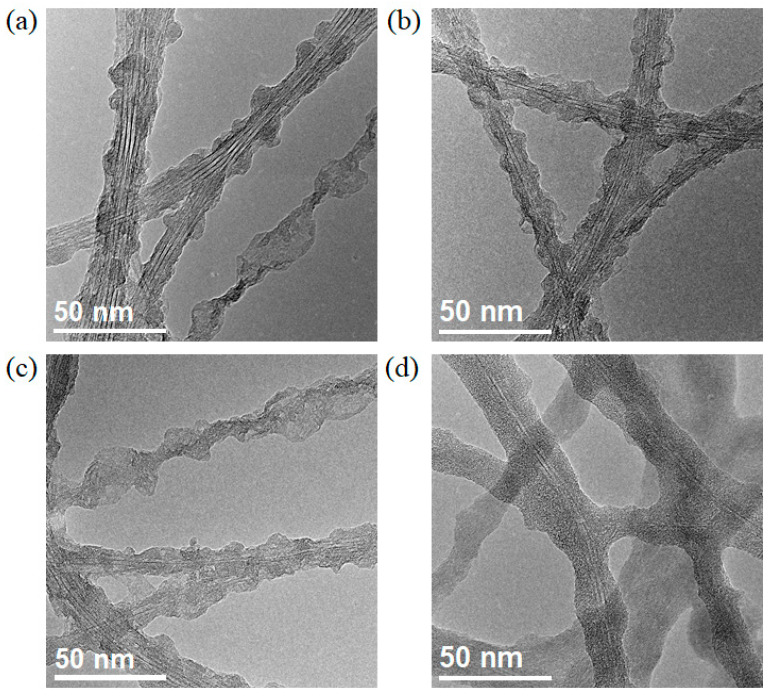
TEM images of the SWCNT@h-BN synthesized using SWCNTs experienced different plasma-treatment time as a template: (**a**) 20 s, (**b**) 40 s, (**c**) 60 s, and (**d**) 100 s.

Since h-BN nucleates from the defect sites on the wall of SWCNTs, we tried to prepare SWCNT@h-BN film with different microstructures by tuning the defect density. SWCNT films were treated by air plasma for different time of 20 s, 40 s, 60 s, and 100 s with a power of 7 W. Upon prolonged treatment time, more defects were generated on the wall of SWCNTs (Figure S8). Then, CVD was performed by using the defective SWCNTs as “templates”, and SWCNT@h-BN with different microstructures were obtained (Figure 5a–d). When a short-time plasma treatment was performed, few defects were generated on the wall of SWCNTs, and limited nucleation sites were provided for h-BN nucleation, which lead to the varying thickness and poor crystallinity of the porous BN layer formed on SWCNTs (Figure 5a). By prolonging the plasma treatment time, more defects were introduced on the wall of SWCNTs, and the thickness uniformity and crystallinity of the h-BN were improved significantly. This can be attributed to the abundant active sites for efficient nucleation and growth of BN. When the plasma time was 100 s, the BN coating became much more uniform (Figure 5d). However, in this case, partial SWCNTs were destroyed during the long-time plasma treatment.

## 4. Conclusions

In summary, SWCNT@h-BN thin films were prepared using SWCNTs with defects as a template. Air plasma treatment was performed to introduce defects that acted as nucleation sites for the CVD growth of h-BN. Combined experiments and first-principles calculations revealed a defect-induced heteroepitaxial growth mechanism of the SWCNT@h-BN. The density of defects on the wall of SWCNTs could be tuned by adjusting the plasma treatment time, and SWCNT@h-BN films with different microstructures were obtained controllably. This work demonstrated that SWCNTs with defects are a promising template for building SWCNT-based derivatives efficiently.

## Data Availability

Data are available from the corresponding author upon reasonable request.

## Supplementary Materials

The following supporting information can be downloaded at: https://www.mdpi.com/article/10.3390/ma16051864/s1, Figure S1. Schematic of the APCVD apparatus used to synthesize SWCNT@hBN; Figure S2. (a) RBM Raman spectra of pristine SWCNTs, plasma-treated SWCNTs, and SWCNT@h-BN, (b) D band Raman spectrum of SWCNT@h-BN excited by 532 nm laser; Figure S3. XPS spectra of SWCNT@h-BN. (a) C 1s, (b) B 1s, and (c) N 1s; Figure S4. TEM images of (a) SWCNT film after 60 s-plasma treatment, (b) SWCNT@h-BN film obtained using SWCNTs shown in (a) as a template; Figure S5. TEM images of the SWCNT@h-BN were synthesized at different temperatures of (a)900 °C, (b) 1000 °C, and (c) 1100 °C.; Figure S6. TEM images of SWCNT@h-BN grown from SWCNTs without plasma treatment; Figure S7. TEM images of the SWCNT@h-BN experienced growth times of (a) 5 min, (b) 15 min, (c) 30 min, and (d) 120 min; Figure S8. D and G bands Raman spectra of the SWCNT@h-BN synthesized using SWCNT templates experienced different plasma treatment time as a template.
