# Supplementary material for: Defect-Induced Efficient Heteroepitaxial Growth of Single-Wall Carbon Nanotubes @ Hexagonal Boron Nitride Films
Source: Materials (Basel). 2023 Feb 24;16(5):1864. doi: 10.3390/ma16051864 (PMC10004222; doi:10.3390/ma16051864)
Supplement: Supplementary file 1 [file materials-16-01864-s001.zip › materials-2225420-supplementary.pdf]

Defect-Induced Efficient Heteroepitaxial Growth of Single-Wall Carbon Nanotubes @ Hexagonal Boron Nitride Films

Changping Yu [1,2], Lili Zhang [1,2], Gang Zhou [3], Feng Zhang [1,2,*], Zichu Zhang [1,2], Anping Wu [1], Pengxiang Hou [1,2], Huiming Cheng [1,4] and Chang Liu [1,2,*]

SUPPLEMENTARY MATERIAL

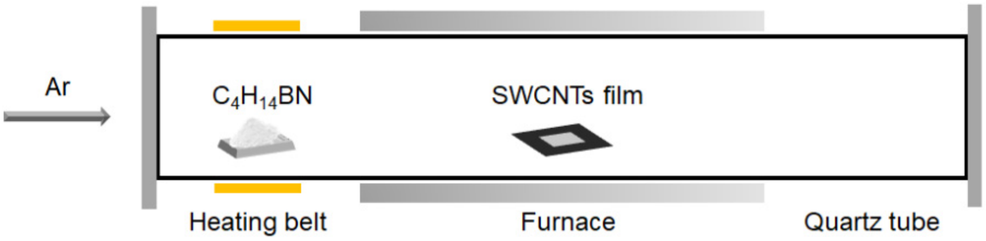

Figure S1. Schematic of the APCVD apparatus used to synthesize SWCNT@h-BN.

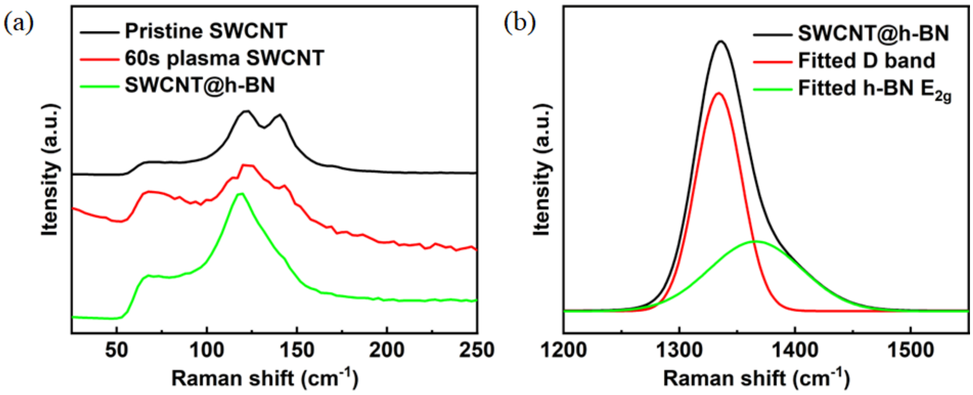

Figure S2. (a) RBM Raman spectra of pristine SWCNTs, plasma-treated SWCNTs, and SWCNT@h-BN, (b) D band Raman spectrum of SWCNT@h-BN excited by 532 nm laser.

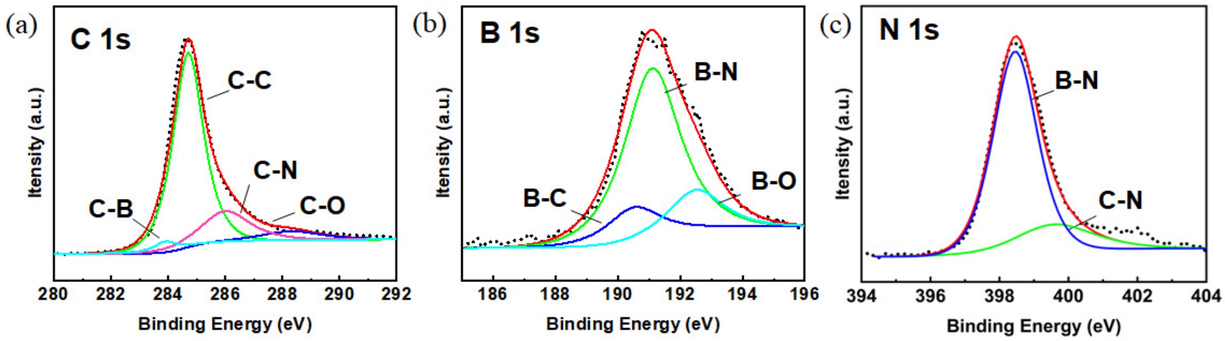

Figure S3. XPS spectra of SWCNT@h-BN. (a) C 1s, (b) B 1s, and (c) N 1s.

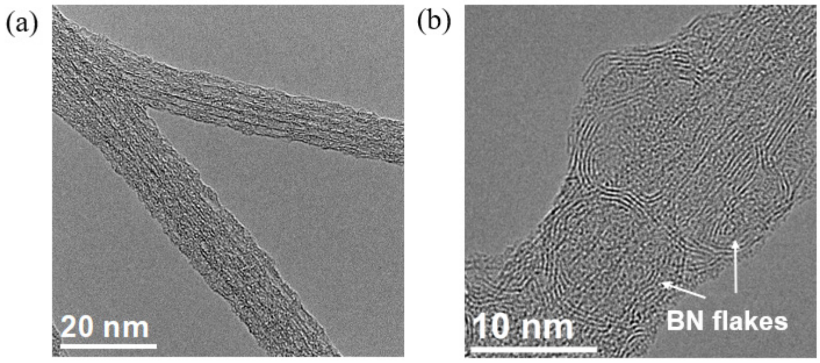

Figure S4. TEM images of (a) SWCNT film after 60 s-plasma treatment, (b) SWCNT@h-BN film obtained using SWCNTs shown in (a) as a template.

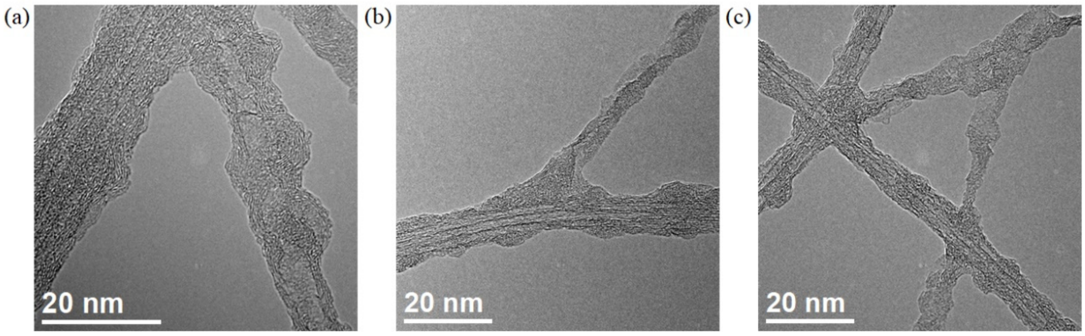

Figure S5. TEM images of the SWCNT@h-BN were synthesized at different temperatures of (a)900 °C, (b) 1000 °C, and (c) 1100 °C.

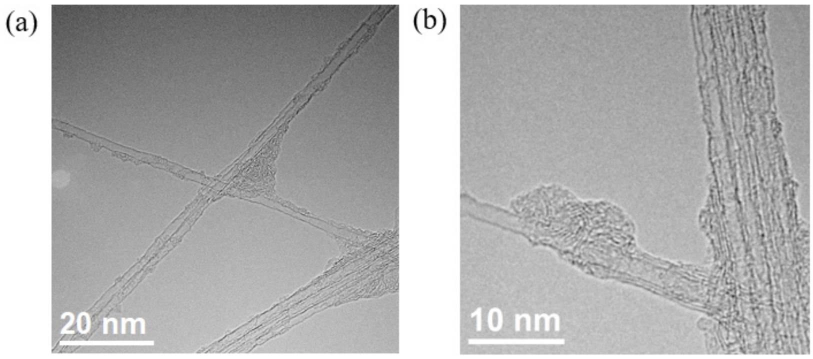

Figure S6. TEM images of SWCNT@h-BN grown from SWCNTs without plasma treatment.

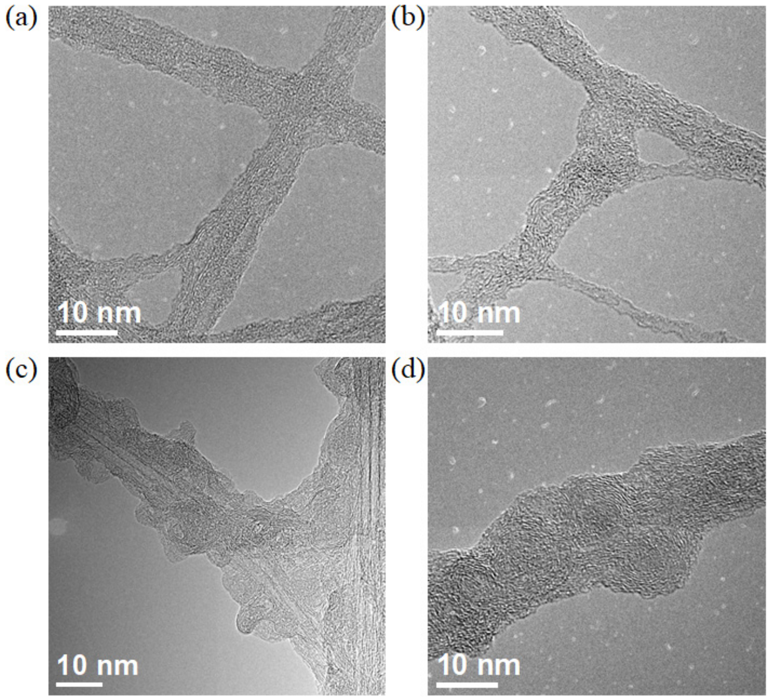

Figure S7. TEM images of the SWCNT@h-BN experienced growth times of (a) 5 min, (b) 15 min, (c) 30 min, and (d) 120 min.

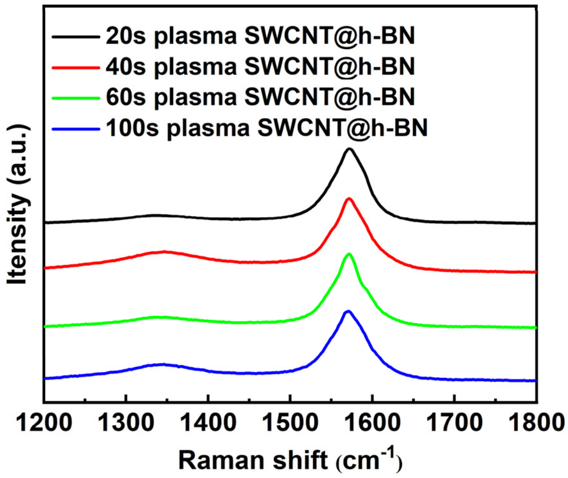

Figure S8. D and G bands Raman spectra of the SWCNT@h-BN synthesized using SWCNT templates experienced different plasma treatment time as a template.
